# N6-methyladenosine RNA methylation regulator-related alternative splicing gene signature as prognostic predictor and in immune microenvironment characterization of patients with low-grade glioma

**DOI:** 10.3389/fgene.2022.872186

**Published:** 2022-07-22

**Authors:** Aierpati Maimaiti, Abudireheman Tuersunniyazi, Xianghong Meng, Yinan Pei, Wenyu Ji, Zhaohai Feng, Lei Jiang, Zengliang Wang, Maimaitijiang Kasimu, Yongxin Wang, Xin Shi

**Affiliations:** ^1^ Department of Neurosurgery, Neurosurgery Centre, The First Affiliated Hospital of Xinjiang Medical University, Urumqi, China; ^2^ Department of Neurosurgery, Xinjiang Production and Construction Corps Hospital, Urumqi, China; ^3^ Department of Neurosurgery, Shenzhen University General Hospital and Shenzhen University Clinical Medical Academy Centre, Shenzhen University, Shenzhen, China

**Keywords:** low-grade glioma, alternative splicing, machine learning, tumor immune microenvironment, prognosis, gene signature

## Abstract

**Background:** N6-methyladenosine (m6A) RNA methylation is an important epigenetic modification affecting alternative splicing (AS) patterns of genes to regulate gene expression. AS drives protein diversity and its imbalance may be an important factor in tumorigenesis. However, the clinical significance of m6A RNA methylation regulator-related AS in the tumor microenvironment has not been investigated in low-grade glioma (LGG).

**Methods:** We used 12 m6A methylation modulatory genes (*WTAP*, *FTO*, *HNRNPC*, *YTHDF2*, *YTHDF1*, *YTHDC2*, *ALKBH5*, *YTHDC1*, *ZC3H13*, *RBM15*, *METTL14*, and *METTL3*) from The Cancer Genome Atlas (TCGA) database as well as the TCGA-LGG (*n* = 502) dataset of AS events and transcriptome data. These data were downloaded and subjected to machine learning, bioinformatics, and statistical analyses, including gene ontology (GO) and the Kyoto Encyclopedia of Genes and Genomes (KEGG) pathway enrichment analysis. Univariate Cox, the Least Absolute Shrinkage and Selection Operator (LASSO), and multivariable Cox regression were used to develop prognostic characteristics. Prognostic values were validated using Kaplan-Maier survival analysis, proportional risk models, ROC curves, and nomograms. The ESTIMATE package, TIMER database, CIBERSORT method, and ssGSEA algorithm in the R package were utilized to explore the role of the immune microenvironment in LGG. Lastly, an AS-splicing factor (SF) regulatory network was examined in the case of considering the role of SFs in regulating AS events.

**Results:** An aggregate of 3,272 *m6A* regulator-related AS events in patients with LGG were screened using six machine learning algorithms. We developed eight AS prognostic characteristics based on splice subtypes, which showed an excellent prognostic prediction performance. Furthermore, quantitative prognostic nomograms were developed and showed strong validity in prognostic prediction. In addition, prognostic signatures were substantially associated with tumor immune microenvironment diversity, ICB-related genes, and infiltration status of immune cell subtypes. Specifically, UGP2 has better promise as a prognostic factor for LGG. Finally, splicing regulatory networks revealed the potential functions of SFs.

**Conclusion:** The present research offers a novel perspective on the role of AS in m6A methylation. We reveal that m6A methylation regulator-related AS events can mediate tumor progression through the immune-microenvironment, which could serve as a viable biological marker for clinical stratification of patients with LGG so as to optimize treatment regimens.

## 1 Introduction

Low-grade glioma (LGG) is the most prevalent type of progressive and aggressive brain cancer affecting ∼5,000 adults in the United States annually (Li G. et al., 2021). LGG is a heterogeneous group of neuroepithelial tumors derived from the malignant transformation of astrocytes or oligodendrocytes (Sun et al., 2021). According to the criteria of the World Health Organization (WHO), LGC can be classified into grade II (diffuse low grade) and grade III (intermediate grade) glioma tumors. Although the clinical outcome of LGGs is relatively good compared to grade IV tumors, the survival of patients among those with LGGs ranges between 1 and 15 years (Gargini et al., 2020). However, 70% of patients with LGGs experience high-grade gliomas, recurrence, and death within 10 years (Van Den Bent, 2014). Long-term survival of patients with LGGs is not only dependent on histological presentation, the extent of resection, and radiotherapy status but also a myriad of molecular features. These include isocitrate dehydrogenase (IDH) 1 and 2 mutations, 1p19q coding deletion, chromosome 10 loss, chromosome 7 gain, chromosome 19/20 co-gain, as well as mutations in *ATRX*, *TP53*, *EGFR*, and *PTEN* (Louis et al., 2021). Nonetheless, as the clinical characteristics of patients with LGGs differ considerably, the progression-free survival (PFS) and overall survival (OS) are significantly diverse, posing a challenge to reliably forecasting prognosis. Thus, a thorough investigation of the modulatory processes of the occurrence and progression of LGG is required to identify biological markers for diagnosis, prognosis, and treatment target identification.

Generally, LGG is characterized by epigenetic alteration that demonstrates substantial genetic and phenotypic variability (Condelli et al., 2019). Traditional epigenetic studies have focused on non-coding RNAs, chromatin remodeling, histone modifications, and DNA methylation (Xiang et al., 2020). In recent years, various reversible chemical changes of RNA have increasingly received attention as a new epigenetic modality of regulation (Cui et al., 2021). Internal modification of RNA by N6-methyladenosine (m6A) is thought to be the most prevalent, pervasive, and maintained alteration of RNA in nature. Since the discovery of RNA demethylases and the advancement of RNA methylation sequencing methods, RNA methylation has been recognized as a common phenomenon (Huang et al., 2020) and a critical modulator of RNA translation, stability, alternative splicing (AS), processing, as well as transcription (Lichinchi et al., 2016). The m6A modification predominantly takes place on the adenine of the RRACH motif sequence according to three protein complexes known as the “writer”, “eraser”, and “reader” (Fu et al., 2014). The encoder (writer) is a methyltransferase, and the components of this complex are known to be METTL3, METTL14, WTAP, ZC3H13, and RBM15; while FTO and ALKBH5 serve as demethylases (eraser) to revert methylation; moreover, the recognition of m6A is accomplished by m6A-binding proteins (readers) found to be YTH structural domain proteins (i.e., YTHDF2, YTHDF1, YTHDC2, and YTHDC1) and the HNRNP family of nuclear inhomogeneous proteins (HNRNPC) (Bian et al., 2020).

The encoder modulates the buildup of the m6A function, whereas the decoder modulates its depletion. Encoders and decoders are essential for the maintenance of a dynamic equilibrium of the levels of m6A in body cells and tissues. In view of the identification of m6A deposits on natural RNA transcripts in the transcriptional process by readers (m6A binding proteins), they could affect post-transcription gene regulation. The abundance and expression of *m6A* regulators are generally dysregulated in a variety of cancers, and crucial for the incidence, progression, metastases, recurrence of cancers, and the development of drug resistance in gliomas (Du et al., 2021). For example, METTL3-mediated m6A alterations were found to be remarkably increased in GBM cells that were resistant to temozolomide. Furthermore, the functional overexpression of *METTL3* in GBM cells can lead to decreased temozolomide responsiveness (Shi et al., 2021). It was also found that YTHDF2 promotes the decay of UBXN1 mRNA through recognition of METTL3-mediated m6A modifications, thereby activating NF-κB and promoting the malignant progression of glioma (Chai et al., 2021). In addition, METTL3 promotes malignant growth in IDH wild-type gliomas via the mechanism of enhancing MALAT1 stability through m6A alteration with the aid of HuR and by means of activating NF-κB. (Chang et al., 2021). It was also discovered that the Jumonji domain-containing 1C (JMJD1C) protein promoted H3K9me1 demethylation in the microRNA miR-302a promoter region, leading to an elevated level of miR-302a expression. MiR-302a has been discovered as a target of the transcription factor METTL3, which can suppress SOCS2 production by modifying the *m6A* gene (Zhong et al., 2021). Furthermore, research on the *m6A* modulatory genes has revealed that they serve as mRNA splicing factors (SFs) for AS and that the *m6A* genes that participate in the regulation mechanism could interface with AS processes as well. AS events are often observed in human cancer cells, and these events are regulated by *m6A* modulators. For instance, in pancreatic ductal adenocarcinoma, HNRNPC inhibits m6A-dependent antimetastatic AS events (Huang et al., 2021). In the case where the CLK1/SRSF5 pathway is activated, aberrant exon skipping in the *METTL14* and *Cyclin-L2* genes are induced, triggering pancreatic ductal adenocarcinoma cell proliferation and metastasis while also modulating m6A methylation (Chen S. et al., 2021). The overexpression of SFs in normal cells could result in the generation of particular pro-oncogenic splice isoforms, thus contributing to the occurrence and progression of cancer.

In post-transcription modulation, AS is among the most essential processes. It is also a regulatory process in which the RNA antecedents are preferentially spliced and ligated. Moreover, it has the potential to produce extensive biodiversity (Cai et al., 2019). The assembling of spliceosomes on pre-mRNA is normally regulated by the SF and the integration of certain exons into the mRNA. As a consequence, under several AS modes (i.e., mutually exclusive exons [ME], alternate terminator [AT], alternate acceptor site [AA], alternate promoter [AP], alternate donor site [AD], retained intron [RI], and exon skip [ES]), complete exons may be spliced into mRNA or be omitted (Li X. et al., 2021). However, in pathological conditions, variable splicing of transcripts could result in functional and structural variability of proteins. Among them, several transcripts might serve as potential tumorigenesis-inducing drivers (Siva et al., 2014; Papatsirou et al., 2021). An additional characteristic of cancer is the expression of splice isomers that are not balanced, or the inability to express the right isomer (Wollscheid et al., 2016; Xiong et al., 2018). A recent research report described that the m6A RNA methylation regulator-associated AS gene signature has the potential to anticipate prognosis in non-small cell lung cancer. They further identified different AS events and their potential regulatory mechanisms to expand further comprehension of tumors in transcriptomic mechanisms. More importantly, there is growing evidence that AS performs an instrumental function in the establishment of the immune microenvironment (Li Z. et al., 2019; Deng et al., 2021). Alterations in AS not only influence the infiltration of immune cells but also modulate tumor-related immune cytostatic functions (Yu et al., 2021).

Although AS is critical in regulating m6A methylation, the clinical significance of *m6A* regulator-associated AS in the tumor microenvironment (TME) has not been investigated in LGG. In the research, we study might offer a new perspective for the identification of biological markers predicting LGG prognosis and explore their prognostic significance for patients with LGG. Finally, our exploration of the mechanism of m6A methylation-related AS mediating tumor progression in the TME could offer a novel insight into its impact on LGG prognosis.

## 2 Materials and Methods

### Data collection and analysis

We queried and acquired gene expression patterns of LGG tissue samples from The Cancer Genome Atlas (TCGA) database (https://portal.gdc.cancer.gov/) for analysis. The relevant clinical-pathological data and Genotype-Tissue Expression (GTEx) data in normal tissues were subsequently obtained from the University of California, Santa Cruz database (UCSC, https://xena.ucsc.edu/), which comprised of 529 LGG tissue samples and 1,152 normal samples (containing 103 normal cerebral cortexes). We did not include patients having inadequate clinical records and lacking follow-up duration information. In this way, a total of 502 LGG samples were included in the present research. In addition, AS events in LGG were downloaded from TCGA Splice Seq (https://bioinformatics.mdanderson.org/TCGASpliceSeq/PSIdownload) and afterward the percentage splicing index (PSI) value, which is a quantitative marker of AS, was calculated, after comparison between subgroups of single and multiple samples, i.e., calculating the percentage value of each AS event, which is commonly utilized in the quantification of AS events. Specifically, for every splicing event in a separate gene, the PSI value is defined as a ratio of the standardized read tally denoting the presence of a transcription component to the overall number of standardized reads (including inclusion and exclusion reads) for that event, with a quantified interval (0–1). Our download includes seven major AS types, namely ME, AT, AP, AA, AD, RI, and ES.

### Identification and profiling of m6A RNA methylation regulatory genes

In total, 12 m6A RNA methylation modulatory genes were chosen for the present research, including 5 “writer”-methyltransferases including *METTL3*, *METTL14*, *WTAP*,*ZC3H13*,*RBM15*, and 2 “writer”-methyltransferases demethylases including m6A demethylase *ALKBH5* and fat mass and FTO, 5 “reader”-binding proteins in the cell including *YTHDC1*,*YTHDC2*,*YTHDF1*,*YTHDF2*, and *HNRNPC*. For this purpose, we input the data of these *m6A* modulators into Cytoscape (version; 3.9.0) and then performed data analysis with the aid of the ClueGO plugin. We then assessed their function in diagnosis, progression prediction, and patient prognosis of LGG. Afterward, we conducted the GO and KEGG pathway and network analyses using the GO terms biological process (BP), molecular function (MF), and cellular component (CC) with adjusted *p* < 0.05 as the threshold of statistical significance. Afterward, we selected and analyzed the differential expression of m6A RNA methylation-regulated genes in LGG tissue samples with the aid of the “LIMMA” R package with the threshold value of |log2 fold-change (FC)| ≥1 and an adjusted *p* < 0.05. Tumor samples were clustered using the R packages “euclidean” and “ward.D2” methods, and the findings of clustering analysis were illustrated by performing the differential expression analysis by virtue of the “pheatmap” R function. An investigation on the relationship between clustering and clinical features was performed by Spearman correlation analysis. With the assistance of the “surv” R package, we conducted univariate and multivariate Cox regression analyses for the purpose of anticipating the correlation between all these m6A RNA methylation-regulated genes and the overall survival of patients.

### Machine learning analysis

We acquired an aggregate of 48,050 AS event features of patients with LGG from the TCGA database using Splice-Seq data. Since the number of features is too large and contains a lot of redundant information causing wastage of computational resources, we used a machine learning method to extract key features. We used AS events to construct the machine learning model and train the model by minimizing the following equation:

min loss function(pred, label)

s.t. data, label

pred=model(data)

In which six machine learning models were “boosting”, “bagging”, “XGBoost”, “Adaboost”, “GBDT”, and “randomforest”. where data ϵ  *R* ^m*n^ is the AS event data, *m* is the number of data, *n* is the dimensionality of the characteristic, pred ϵ  *R* ^m*n^ is the results of AS event data predicted with the aid of machine learning models, *loss_function* denotes the loss function, and the difference between *pred* and *label* is measured with second-order Euclidean distance as the loss function so as to optimize the model, achieving an accuracy rate of over 85%. Thus, we extracted the key features of each model, sorted their importance, and observed the prediction effect of the model under the different number of features. Finally, the features are screened as effective features when the prediction effect is stable.

### Identification of gene AS events with OS of patients with LGG

We used the “WGCNA” (Weighted gene co-expression network analysis) R software package to associate AS events with OS in patients with LGG. WGCNA is capable of analyzing a large amount of genomic data to discover genomes correlated with tumor phenotypes. Hence, it enabled us to process data on AS events from clinical features and *m6A* modulatory gene expression profile association information, while avoiding the use of redundant numerous hypothesis tests and correction procedures. For this purpose, we obtained the AS event data according to the formula *A*_*ij*_ = power (*S*_*ij*_, *β*) = |*S*_*mn*_|*β* (*j* and *i* denote the AS event of *j* and *i*, respectively, whereas *n* and *m* denote the node connections counts, and *β* denote the appropriate soft threshold power) was computed for the standard scale-free network to produce the appropriate value of *β*. Then, we used the topological overlap matrix *TOMij=* $\frac{\sum_{u}AiuAju+Aij}{\min\left(Ki,Kj\right)+1-Aij}$ (*i* and *j* denote the AS event associated with *i* and *j*, respectively, whereas *u* denotes clinical characteristics and prognosis data)*.* This step involves the establishment of a weighted adjacency matrix, which was translated in the form of TOM. Subsequently, we employed a dynamic tree cutting approach for the purpose of finding the modules that are strongly correlated with AS events on the basis of hierarchical clustering. 1-TOM was used as the AS event distance measure for depth (threshold value of 2) and least size (threshold value of 60). Thereafter, relatively identical modules were merged with the aid of clustering and a height threshold of 0.3 that was determined by earlier research. Eventually, we carried out Spearman correlation as well as module signature gene analyses on the expression of the *m6A* modulator gene for the purpose of investigating the correlation between clinical characteristics and prognosis of 502 patients with LGG. Considering previous research, we identified and examined the modules that were most remarkably correlated with clinical characteristics and *m6A* modulator genes, i.e., an absolute correlation coefficient between *m6A* regulator and AS event modules exceeding 0.4 with adjusted *p* < 0.05. Finally, we conducted univariate and multivariate Cox regression analyses to examine the correlation between AS events and the OS of patients with LGG.

### GO and KEGG enrichment analysis of AS genes

Following the screening for AS events related to m6A, we analyzed these OS-associated AS genes using GO terms (CC, MF, and BP) and KEGG pathway analyses. The data for *m6A-related* AS genes and *m6A*-modulated genes were further explored with the aid of the ClueGO plugin of Cytoscape. In order to determine the threshold for statistical significance, an adjusted *p* < 0.05 was used. Following that, we constructed functional networks for the associated genes using Cytoscape.

### Establishment and evaluation of the m6A RNA methylation regulator-related AS gene risk model

To discover *m6A* regulator-related AS events associated with survival, we performed univariate Cox regression analysis to delve into the correlation between *m6A* regulator-associated AS events and the OS of patients with LGG (*p* < 0.05). Then, the obtained data were subjected to visualization with the aid of volcano and UpSet plots. In addition, bubble plots were drawn to demonstrate the 20 most significant AS events across the seven classes of survival-associated AS events. Second, for the purpose of screening candidates within every clipping sequence and preventing model overfitting, we conducted minimal Lasso regression analysis. Ultimately, the seven AS events were integrated into the multi-factor Cox regression analysis with the aim of identifying prognosis predictors. Moreover, prognosis risk scores for predicting OS were produced using the seven AS events. The PSI values of the AS events were utilized to develop a multivariate prognostic model. The formula below was utilized to derive the risk scores:

$$\mathrm{Risk score}=\;\sum_{\text{i}=1}^{n}\beta_{i}\times \mathrm{PSI}$$

where *n* denotes the number of survival-associated AS events, *βi* denotes the coefficient index of those events, and *PSI* denotes the PSI value of those events. Based on the median value of the risk scores, the LGG sample was classified into two groups, namely the high- and low-risk groups. To contrast the survival rates between the two groups, we employed the Kaplan-Meier survival curves. Moreover, a two-sided log-rank test, as well as the computation of the area under the curve (AUC), were performed with the aid of the “survivor” and “survminer” R packages. The significance criterion was set as *p* < 0.05. In addition, with the help of the R package “survival ROC” software, we successfully generate time-dependent receiver operating characteristic (ROC) curves to investigate the prognostic significance of this characteristic.

### Examination of independent prognostic significance of m6A RNA methylation regulator-related AS risk score

For the purpose of determining if risk scores independently served as an indicator of prognosis, univariate and multivariate Cox regression analyses were performed utilizing risk values and accessible clinical data acquired from TCGA cohort.

### Nomogram as a prognostic model based on AS events and clinicopathological characteristics

The model incorporated risk scores for *m6A* modulator-related AS events in addition to the clinical indicators of LGG, including age, gender, grading, diagnosis, and type (primary and recurrent), as well as mutation status of *IDH1* (*R132*), *ATRX*, *TP53*, *EGFR*, and *PTEN*. This allowed the optimization of its prediction power, and the “rms” R package was used to determine independent prognostic factors, while relevant clinical parameters in the TCGA cohort were used as variables for the construction of column line plots. We created a horizontal straight line for the purpose of denoting the locations of the point of each variable according to the number of variables. Furthermore, the total number of points for each patient was obtained by adding all the points of the variables and standardizing their distribution between 0 and 100. OS values were calculated for patients with LGGs at 1, 2, and 3 years by placing them between each prognostic axis and the total points axis. Subsequently, we drew calibration plots with the aid of the “rms” R package, and the “rmda” and “devtools” R packages were applied to perform clinical decision curve analysis (DCA) to validate the utility of the column line plots in the cohort.

### Relationship between risk score and characterization of tumor-infiltrating immune cells

We obtained information on the immune infiltration of each sample (including neutrophils, CD8^+^ T cells, macrophages, dendritic cells, CD4^+^ T cells, and B cells) using Tumor Immune Evaluation Resource (TIMER) (https://cistrome.shinyapps.io/timer/) (TIMER). Then, we probed into the correlation between tumor immune cell (TIC) infiltration and prognosis risk scores. We used the “GSEAbase” R package to perform a single-sample gene set enrichment analysis (ssGSEA) of the enrichment of two separate risk groups in a set of 29 immune function-related genes. Subsequently, the “ESTIMATE” R package was used with the aim of measuring the extent and degree of tumor purity and infiltrated cells (i.e., immune and macrophages cells), thus confirming that the tumor immune milieu features of the two risk groups were significantly different. Identification of cell types was performed based on the levels of 22 distinct types of immune cells present in each tumor sample, which was obtained by measuring the relative subset of RNA transcripts (CIBERSORT; https://cibersort.stanford.edu/).

### Risk score function in immune checkpoint blockade (ICB) therapy

The expression of key genes associated with ICB may correlate with the clinical outcomes following immune checkpoint inhibitor treatment of existing studies. Six critical genes for immune checkpoint blockade treatment, including indoleamine 2,3-dioxygenase 1 (*ID O 1*), cytotoxic T lymphocyte antigen 4 (*CTLA-4*), programmed death-ligand 1 (*PD-L1*, commonly referred to as *CD274*), *PD-L2* (also known as *PDCD1LG2*), *PD-1* (also designated *PDCD1*), and T-cell immunoglobulin structural domain and mucin-containing structural domain molecule-3 (*TIM-3*, also named *HAVCR2*). To reveal possible participants in constructing risk profiles in ICB-treated LGG, AS-based prognostic features were correlated with the expression of the six genes that participate in ICB. Furthermore, we analyzed the expression of 47 genes correlated with ICB in patients belonging to the two groups.

### Correlation between SFs and AS events as well as their modulatory network

SFs can modulate AS events in the TME. As a result, developing a prognosis prediction model founded on AS events calls for simultaneous investigation of the correlation between AS events and SFs. Specifically, the PSI levels of AS events and the expression of potential SFs involved in these events were examined by means of Pearson’s correlation test. Statistical significance was set at *p* < 0.001 and correlation coefficients (*r*) > 0.6 or < –0.6. Subsequently, according to the analytic outcomes, we constructed a regulatory network between SFs and AS events, which was visualized with the aid of Cytoscape.

### Statistical analysis

All statistical analyses were performed with the aid of R (version: 4.0.3). The “UpSetR” R package was utilized to examine the intersects and clusters of a variety of AS events. The “ClusterProfiler” R package was used for the purpose of conducting the KEGG and GO analyses. The “survival” and “survROC” R packages were used to perform the survival analysis. The LASSO multivariate Cox analysis was performed with the “glmnet” R package. The rates of survival were analyzed utilizing Kaplan-Meier curves and log-rank test, thus determining the possible statistical significance. Pearson correlation tests were used to investigate the correlation between risk scores, clinical factors, the extent of immune cell infiltrates, and immunological checkpoints. A criterion of *p* < 0.05 denoted statistical significance.

## 3 Results

### Clinical characteristics and AS event profiles in LGG

We obtained LGG expression profile data from the TCGA database, as well as clinicopathology data from the UCSC database that were used in the present research. We obtained 502 LGG cases for data analysis. For the LGG samples, there were 278 males and 224 females, 232 patients aged <40 years, and 270 individuals aged ≥40 years. The patients were graded as G2 (*n* = 241) and G3 (*n* = 261) (Table 1). We observed 48,050 AS events in the LGG tissue sample, with ES being the most common of all AS events. AP ranked second as the most prevalent, with ME ranking last.

**TABLE 1:** Baseline data of all LGG patients.

Characteristic	Type	n	Proportion (%)
Age	<40	232	46.22
	≥40	270	53.78
Gender	Female	224	44.62
	Male	278	55.38
Grade	G2	241	48.01
	G3	261	51.99
Diagnoses	Astrocytoma, anaplastic	129	25.70
	Astrocytoma, NOS	61	12.15
	Mixed glioma	128	25.50
	Oligodendroglioma, anaplastic	76	15.14
	Oligodendroglioma, NOS	108	21.51
Type	Primary Tumor	483	96.22
	Recurrent Tumor	19	3.78
Chr 19/20 co-gain	Gain chr 19/20	12	2.39
	No chr 19/20 gain	487	97.01
	unknow	3	0.60
Chr 7 gain/Chr 10 loss	Gain chr 7 & loss chr 10	55	10.95
	No combined CNA	444	88.45
	unknow	3	0.60
IDH1 R132status	Mutation	386	76.89
	Wild	116	23.11
PTEN status	Mutation	29	5.78
	Wild	473	94.22
EGFR status	Mutation	30	5.98
	Wild	472	94.02
ATRX status	Mutant	185	36.85
	WT	317	63.15
TP53 status	Mutation	229	45.62
	Wild	273	54.38

### Correlation between m6A RNA methylation regulatory gene expressions and LGG prognosis

**FIGURE 1:**
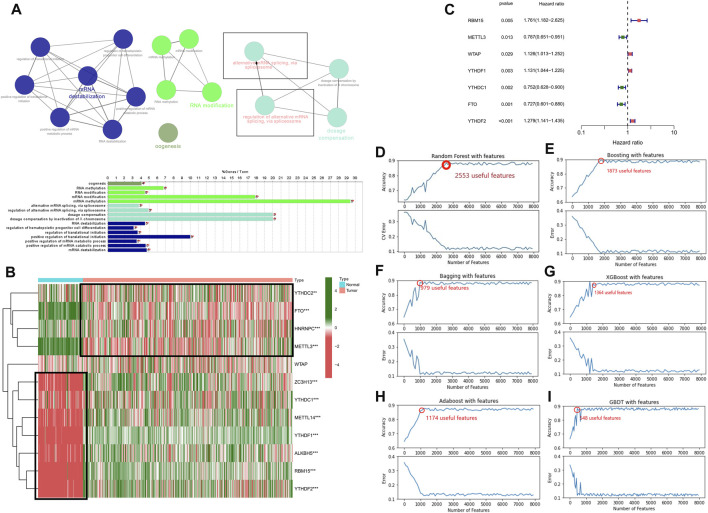
Relationship between the methylation status of the *m6A* RNA modulatory genes and the prognosis of patients with LGG **(A)** The GO terms and KEGG enrichment pathways analyses of the *m6A* modulator genes; the various colors reflect the various pathways. **(B)** In LGG, 12 *m6A* RNA-methylated regulator genes exhibit differential expression **(C)** The forest plot representing the analytical data for the univariate Cox regression. The 12 *m6A* RNA methylation modulators in LGG examined by means of univariate Cox regression and visualized by a forest plot. **(D–I)** Six algorithms applied to screen the potential AS. The number of features with the maximum accuracy and least margin of error after fivefold cross-validation in “Boosting”, “Bagging”, “XGBoost”, “Adaboost”, “GBDT”, and “Randomforest”. ***p < 0.001, **p < 0.01, and *p < 0.05.

In the present research, we examined 12 *m6A* genes that were involved in the regulation of RNA methylation. We carried out GO terminology and KEGG pathway analysis and the findings demonstrated a remarkable enrichment of these modulators of *m6A* in mRNA spliceosome biological processes, RNA modifications (RNA methylation biological processes), and RNA instability biological processes (Figure 1A). In addition, the findings recorded from the Wilcoxon rank-test demonstrated a differential expression in normal and LGG tissues except for *WTAP* (*p* < 0.001) (Figure 1B). The results obtained from the univariate Cox regression illustrated that the expression of *RBM15*, *WTAP*, *YTHDF1*, *METTL3*, *YTHDC1*, *FTO*, and *YTHDF2* predicted OS in patients with LGG (*p* < 0.05) (Figure 1C). Therefore, we included *BM15*, *METTL3*, *YTHDF1*, *YTHDC1*, *FTO*, and *YTHDF2* regulatory genes as subsequent SFs for association analysis.

### Development and verification of the AS events based on multiple machine learning algorithms

We used six machine learning models such as “boosting”, “bagging”, “XGBoost”, “Adaboost”, “GBDT”, and “randomforest” to further elucidate the selected AS events. We used the final merged set of valid features for each of the six models as the variable clipping events for the next task, and a total of 3,272 valid features were extracted for the variable clipping events. We then visualized the features filtered by each machine learning model (Figures 1D–I). After comparison, we found that using the above-combined features, the prediction effect is basically comparable to the full number of features but reduces the number of operations by 90%, thus maximizing the prediction effect under the condition of limited computational resources.

### Identification of m6A RNA methylation regulatory genes with LGG clinical characteristics

**FIGURE 2:**
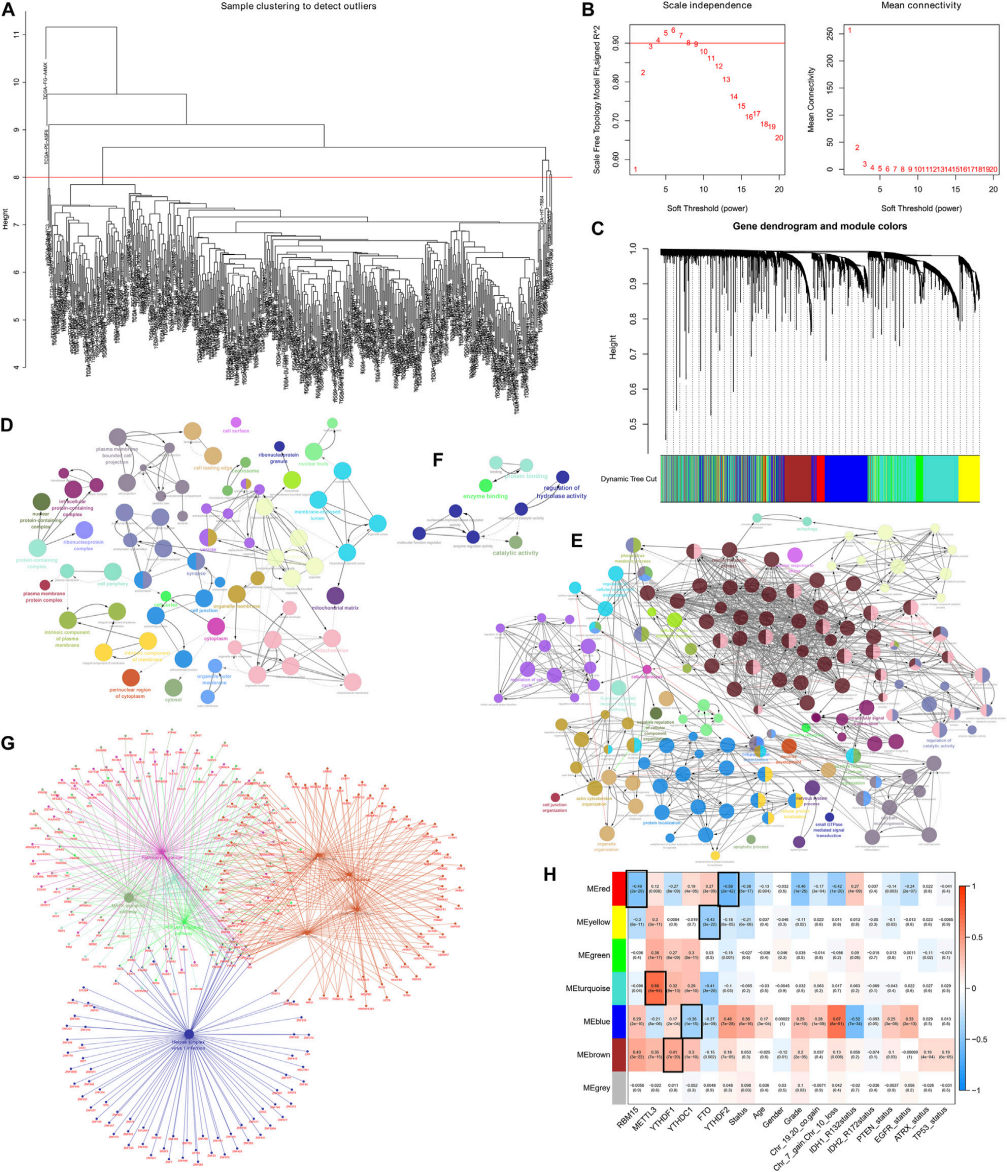
Determination of the AS events that are correlated with clinical characteristics and the *m6A* regulator expression **(A)** Sample dendrogram and trait indicator. **(B)** Assessment of the soft-threshold power of WGCNA analysis **(C)** The AS events in LGG samples are clustered hierarchically. The tree represents an LGG with a distinct name and experiment identification. **(D–G)** Genes associated with m6A-related AS events in LGG were subjected to analysis using GO terms and KEGG pathways. The GO terms **(D–F)** and KEGG pathways enrichment **(G)** analysis of the m6A-related prognostic AS genes in LGG **(H)** Association of the AS events with *m6A* methylation regulatory genes, age, gender, grade, Chr 7 gain/Chr 10 loss, as well as Chr 19/20 co-gain, mutations status of *IDH1* (*R132*), *IDH2* (*R172*), *PTEN*, *EGFR*, *ATRX*, and *TP53*.

In the present research, we examined the relationship between AS events and weighted gene co-expression networks. The results demonstrated that they were congruent to the scale-free network (Figure 2B). The log10 transformed RNA-seq scores were discovered by performing hierarchical cluster analysis of the samples using Euclidean distance (Figure 2A), whereas the dynamic tree cutting technique exposed modules with similar expression patterns and combined similar modules (Figure 2C). We then analyzed AS events using the “WGCNA” R package and linked *m6A* regulator gene expression to clinical traits using Spearman correlation tests.

The findings revealed that the MEyellow module was related to the expression of genes regulating *m6A* in patients with LGG (*RBM15*, *P* = 8e-11, *r* = –0.3; *METTL3*, *P* = 3e-11, *r* = 0.3; *FTO*, *P* = 3e-22, *r* = –0.43; *YTHDF2*, *P* = 8e-05, *r* = –0.18), and also significantly correlated with survival status (*P* = 6e-6, *r* = –0.21), grading (*p* = 0.02, *r* = –0.11), and PTEN mutation status (*p* = 0.03, *r* = –0.1).

In addition, MEred, MEturquoise, MEblue, and MEbrown modules exhibited a remarkable association with the expression of genes regulateing *m6A* (MEred: *RBM15*, adjusted *P* = 2e-29, *r* = –0.49; *METTL3*, adjusted *p* = 0.008, *r* = 0.12; *YTHDF1*, adjusted *P* = 8e-9, *r* = –0.27; *YTHDC1*, adjusted *P* = 4e-5, *r* = 0.19; *FTO*, adjusted *P* = 3e-9, *r* = 0.27; *YTHDF2*, adjusted *P* = 2e-42, *r* = –0.58; MEturquoise: *RBM15 p* = 0.04, *r* = –0.096; *METTL3*, adjusted *P* = 4e-64, *r* = 0.68; *YTHDF1*, adjusted *P* = 9e-13, *r* = 0.32; *YTHDC1*, adjusted *P* = 4e-10, *r* = 0.29; *FTO*, adjusted *P* = 3e-20, *r* = –0.41; *YTHDF2*, adjusted *p* = 0.03, *r* = –0.1; MEblue: *RBM15*, adjusted *P* = 2e-10, *r* = 0.29; *METTL3*, adjusted *P* = 8e-6, *r* = –0.21; *YTHDF1*, adjusted *P* = 2e-4, *r* = 0.17; *YTHDC1*, adjusted *P* = 1e-15, *r* = –0.36; *FTO*, adjusted *P* = 4e-9, *r* = –0.27; *YTHDF2*, adjusted *P* = 7e-28, *r* = 0.48; MEbrown: *RBM15*, adjusted *P* = 3e-22, *r* = 0.43; *METTL3*, adjusted *P* = 7e-15, *r* = 0.35; *YTHDF1*, adjusted *P* = 7e-20, *r* = 0.41; *YTHDC1*, adjusted *P* = 1e-10, *r* = 0.3; *FTO*, adjusted *p* = 0.002, *r* = –0.15; *YTHDF2*, adjusted *P* = 7e-5, *r* = 0.18).

In addition to this, the MEred, MEblue, and MEbrown modules were also correlated with the tumor grade of the patient (MEred: adjusted *P* = 1e-25, *r* = –0.46; MEblue: adjusted *P* = 1e-10, *r* = 0.29; MEbrown: adjusted *P* = 2e-5, *r* = 0.2), Chr 7 gain/Chr 10 loss (MEred: adjusted *P* = 1e-20, *r* = –0.42; MEblue: adjusted *P* = 8e-61, *r* = 0.67; MEbrown: adjusted *p* = 0.006, *r* = 0.13), and PTEN mutation status (MEred: adjusted *p* = 0.003, *r* = –0.14; MEblue: adjusted *P* = 3e-8, *r* = 0.25; MEbrown: adjusted *p* = 0.03, *r* = 0.1) (Figure 2H).

The above findings revealed that m6A-related AS events in the MEbrown, MEblue, and MEred modules forecast LGG progression and tumor grade, whereas the gain or loss of chromosomes and PTEN mutation status of patients may also influence m6A-related AS events. In addition, the MEred module included 208 AS events, the MEyellow and MEbrown modules both 386 AS events, the MEturquoise module 1,181 AS events, and the MEblue module 768 AS events. Analysis of GO terms and KEGG pathways in the LGG cohort showed that AS events associated with *m6A* were significantly enriched in mitogen-activated protein kinase (MAPK) signaling pathway, cancer signaling pathway, PI3K-Akt signaling pathway, neurodegenerative ailments, particularly, Alzheimer’s disease, Huntington’s disease, and processes of the nervous system, cellular metabolic processes, intracellular signaling, cell cycle regulation, regulation of catalytic activity, regulation of hydrolase activity, etc. (Figures 2D–G)

### Identification of m6A-Related AS events with LGG prognosis

**FIGURE 3:**
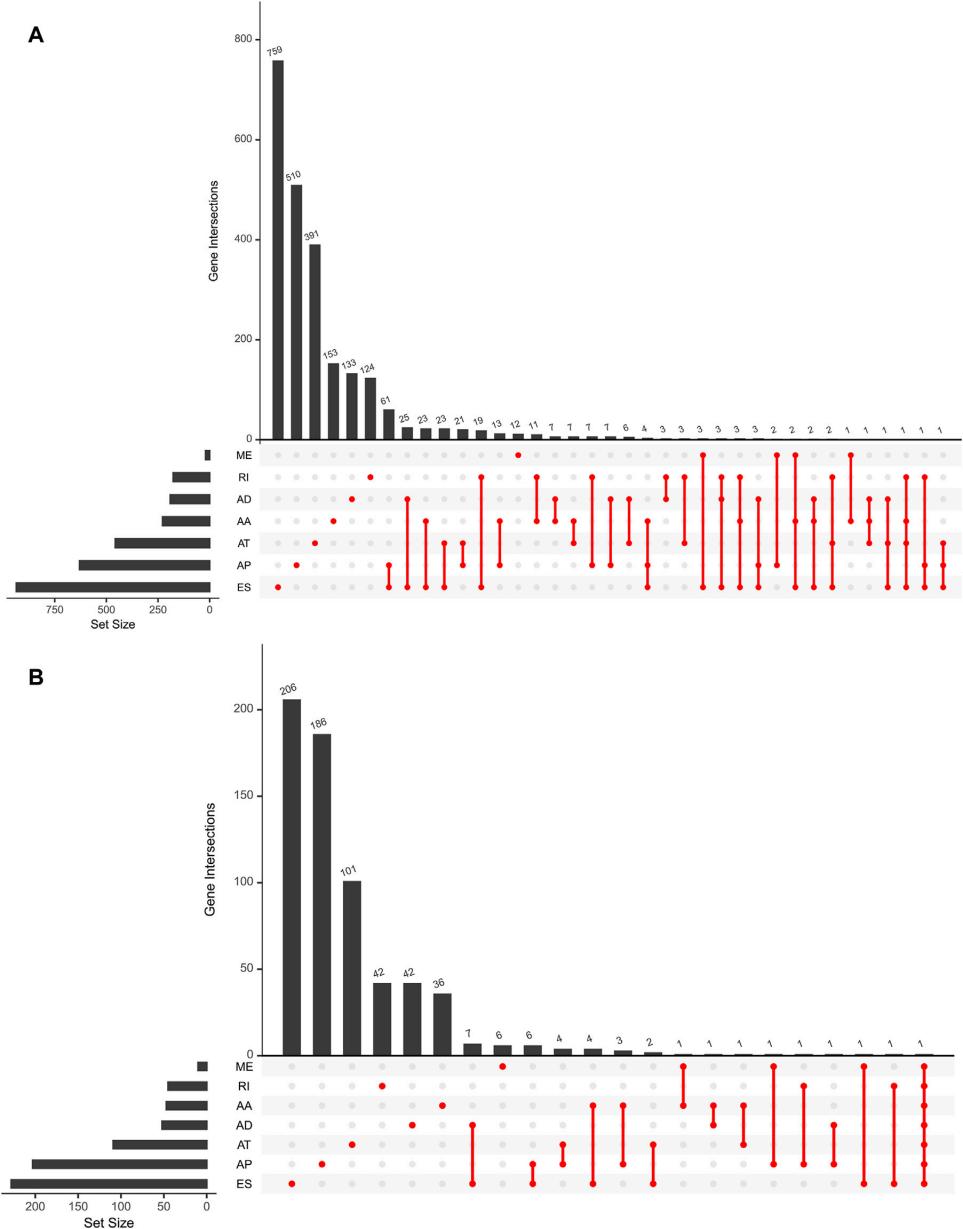
**(A)**. An upset plot depicting interactions between distinct m6A methylation regulator-related AS types In LGG. **(B)**. Upset plot showing survival-associated m6A methylation regulator-related AS types.

**FIGURE 4:**
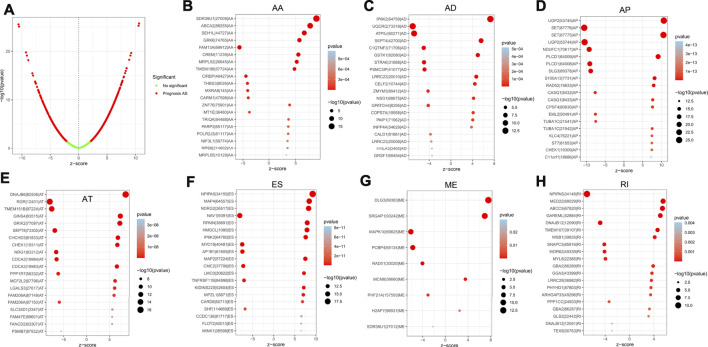
**(A)**. The volcano plots of AS events associated with the m6A methylation regulator that is survival-relevant. In the TCGA-LGG cohort, the most crucial m6A-relevant RIs MEs, ESs, ATs, APs, ADs, and AAs were identified **(B–H)**.

For the purpose of revealing the correlation between m6A-related AS events and LGG prognosis, we conducted a univariate Cox regression analysis of the data. With the aid of the UpSet plot, we identified 750 prognostic AS events in LGG, with intersecting genomic and splice subtypes (*p* < 0.05; Figures 3A,B, Supplementary Table S1). ES was the most prevalent pattern compared with other subtypes of AS events, followed by AP and ME, with ME being the least common pattern. Volcano plots were created for the purpose of visualizing the events of AS (Figure 4A). The 20 key AS events associated with survival in the seven subtypes is summarized in Figures 4B–H.

### Construction and verification of the prognostic risk score model based on m6A-related AS events

**FIGURE 5:**
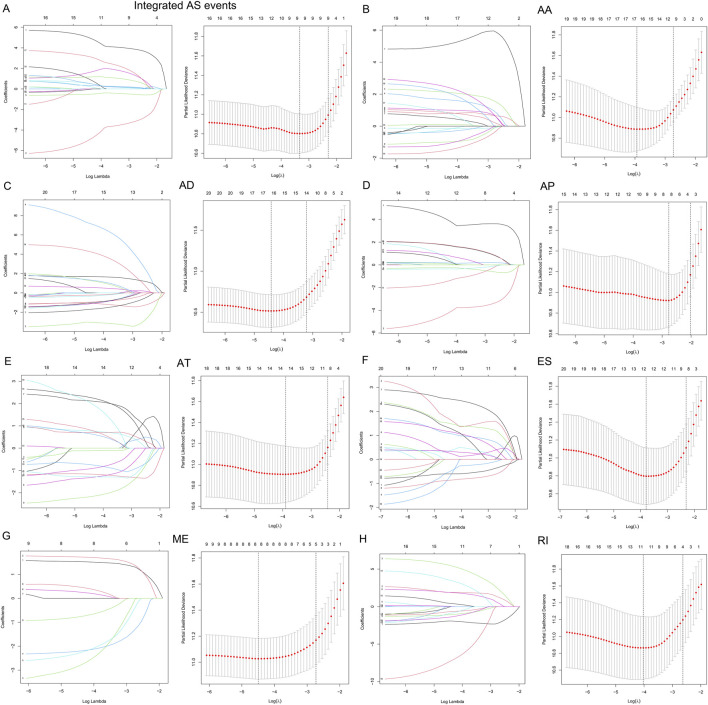
**(A–H)**. AS events associated with the m6A methylation regulator and their correlation with the prognosis of patients with LGG were identified and validated utilizing LASSO regression. The 10-round cross-validation method was used for the purpose of evaluating these 12 m6A-related AS events that were correlated with the prognosis of patients with LGG, as well as the optimum levels of the penalty parameter.

**FIGURE 6:**
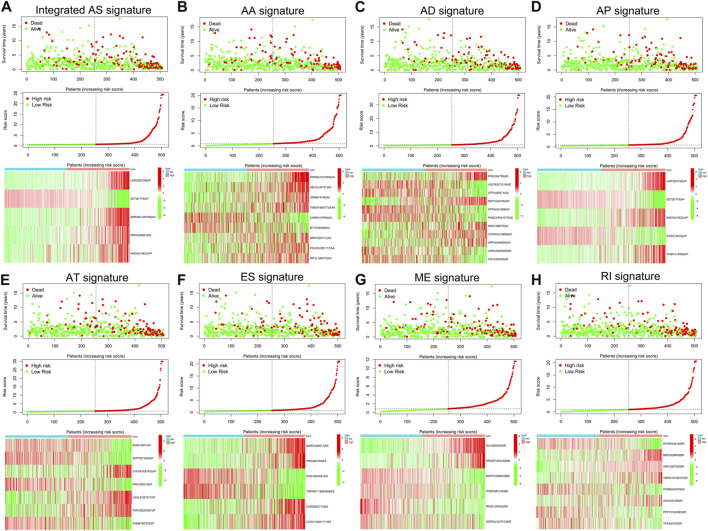
**(A–H)**. The risk scores associated with seven different kinds of m6A methylation regulator-associated AS signatures and combined AS signatures in patients with LGG. The spread of overall survival for patients with different risk scores is shown in the top panel of the figure. The variance trend in patient survival time with risk scores is shown in the center panel. The heatmap of AS events associated with the survival-related m6A methylation modulator is shown in the lower panel.

**FIGURE 7:**
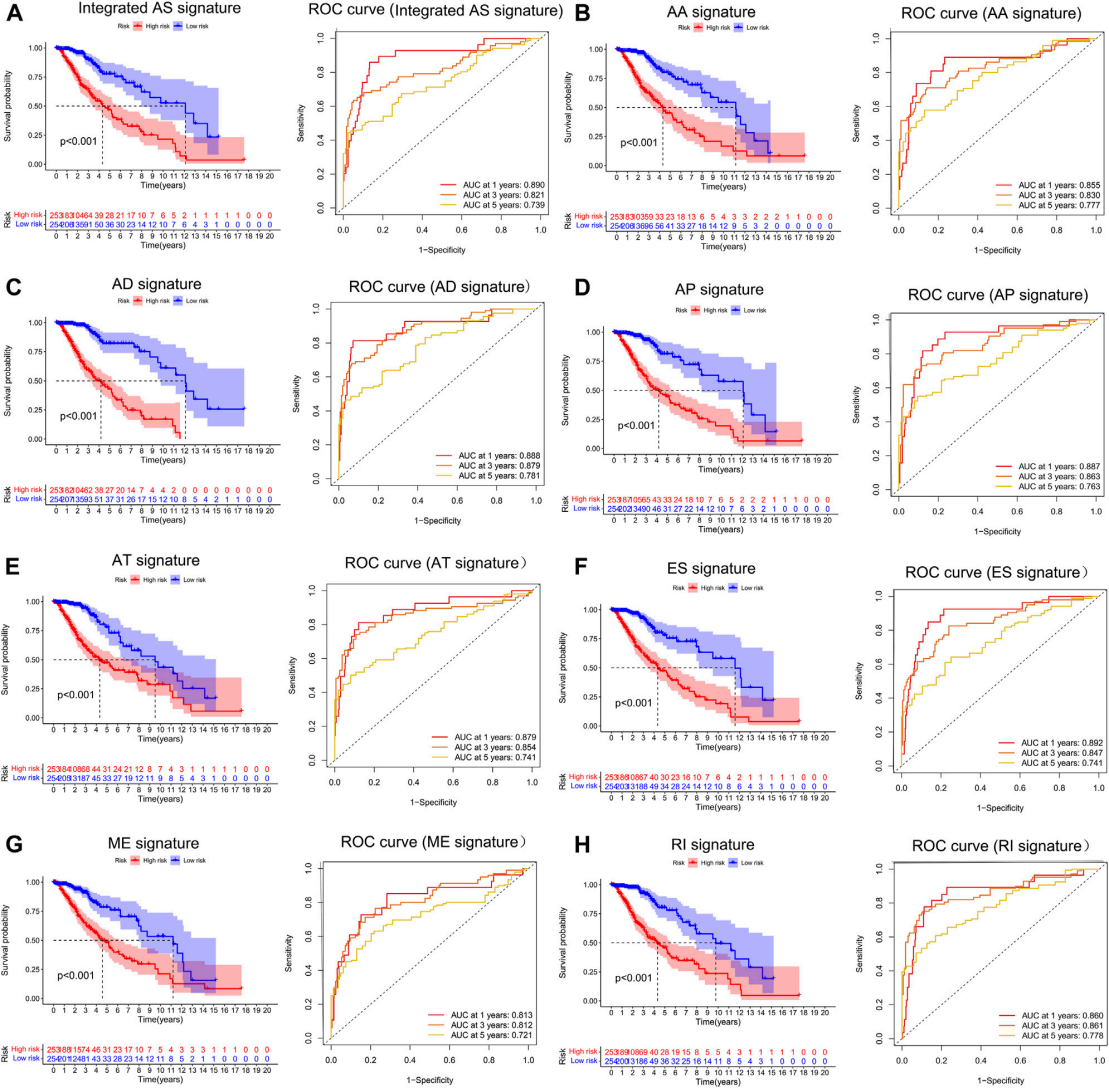
**(A–H)**. Treatment outcome in the LGG cohort determined by survival analysis of the patients using the m6A methylation regulator-related AS signature and the Verification of the predictive efficacy of all AS signatures and the combined AS signature. The Kaplan-Meier survival curves of the patients in the high- and low-risk groups are shown in the left panel. The right panel depicts the ROC curve illustrating the prognostic precision of the individual AS signature and the combined AS signature.

To examine the ability of *m6A* regulator-related AS events in predicting patient prognosis, the above 7 AS events and their combinations were further subjected to LASSO regression and screened for the most important *m6A* regulator-related AS events. Figures 5A–H shows the results of the LASSO regression analysis. A multivariate Cox regression model was then employed on the independent prognostic indicators. A risk score was generated for every patient, and complete details on the prognostic factors based on the seven AS events are provided in Table 2. Patients with LGG were classified into two groups (low- and high-risk groups) according to their risk scores. Figures 6A–H depicts data on the kinds of potential AS events, as well as data on survival time and survival statuses, which are arranged based on the distributions of risk values. Patients in the low-risk group exhibited a more favorable prognosis as opposed to the ones in the high-risk subgroup, according to the findings of the Kaplan-Meier survival analysis (*p* < 0.05, Figures 7A–H). For the purpose of assessing the prognostic accuracy of the variables across time, a time-dependent ROC analysis was performed at 1, 3, and 5 years. In all cases with AS event characteristics, the ROC AUC values were over 0.7, indicating strong prognostic prediction ability (Figures 7A–H). The above findings suggest that the AS-based prognostic signature and risk scoring system has the potential to be used as a new method for LGG classification.

**TABLE 2:** Multivariate Cox analysis of prognostic *N6-Methyladenosine-Related AS* signatures of each AS type.

Type	Gene symbol	Splice-seq CD	AS type	Coef	HR	HR95L	HR95H	P-value
Integrated AS	UGP2	53745	AP	4.879983645	131.6285111	19.19928284	902.432924	6.75E-07
	SET	87776	AP	−4.394829337	0.012340986	0.000431926	0.352606634	0.010187646
	SDR39U1	27009	AA	3.2291677	25.25862547	0.527549531	1209.361629	0.101845454
	RPAIN	38691	ES	3.338637289	28.18069839	3.858762106	205.8047995	0.000998063
	RAD52	19633	AP	1.411443796	4.101873398	0.932391021	18.04539618	0.06185279
AA	SDR39U1	27009	AA	6.084249222	438.8901798	15.58619807	12358.66432	0.000353431
	SEH1L	44727	AA	3.297355611	27.04103728	3.140548345	232.8312182	0.002684096
	GRK6	74765	AA	2.321697441	10.19296158	1.327158745	78.28488208	0.02560975
	TMEM198	57724	AA	1.800566228	6.053073916	0.916948689	39.95829241	0.06149485
	CARM1	47598	AA	−1.506535512	0.221676645	0.071078446	0.69135635	0.009432271
	MT1E	36480	AA	−1.84909159	0.157380067	0.056506714	0.438328189	0.000402925
	PARP3	65117	AA	3.146071769	23.24457494	3.012209603	179.3733953	0.002547726
	POLR2J3	81117	AA	2.046993192	7.7445796	1.760038909	34.07794729	0.006773064
	NIF3L1	56774	AA	3.053794574	21.19562036	4.631422605	97.00136673	8.31E-05
AD	IP6K2	64759	AD	1.922510982	6.838107292	1.876547772	24.91794349	0.003568075
	UQCRQ	73319	AD	−1.17436915	0.30901386	0.092460845	1.032756793	0.056441926
	ATP5J	60271	AD	−3.175511473	0.041772733	0.00084905	2.05519287	0.110141838
	SEPT4	42700	AD	8.554871125	5191.983732	88.70457802	303892.9408	3.79E-05
	STRA6	31688	AD	−2.031010147	0.13120292	0.05125151	0.335877052	2.29E-05
	PSMC3IP	41077	AD	−1.159759969	0.313561436	0.119740086	0.821118285	0.018214528
	NSG1	68675	AD	0.850737895	2.341373902	1.007619205	5.440578862	0.047972912
	COPS7A	19958	AD	5.018203401	151.1395227	20.88297137	1093.865184	6.72E-07
	INPP4A	54629	AD	1.708539296	5.520891197	1.366237351	22.30962254	0.016487465
	LRRC23	20008	AD	−1.553350033	0.211538126	0.053381317	0.838277909	0.027031513
	HHLA3	3405	AD	−1.48302156	0.226950905	0.047902498	1.075240657	0.061684026
AP	UGP2	53745	AP	4.312239301	74.60737036	8.759537721	635.4513091	7.96E-05
	SET	87776	AP	−5.016555712	0.006627314	0.000343099	0.128013404	0.000898039
	RAD52	19633	AP	1.940344746	6.961150388	1.533485812	31.59964985	0.011940996
	CASQ1	8432	AP	−1.87466241	0.153406747	0.01906347	1.234488279	0.078074254
	CHEK1	19309	AP	2.040848212	7.69713523	1.219051932	48.59997282	0.029958873
AT	RGR	12401	AT	−1.52922525	0.216703493	0.065338895	0.718720508	0.01242313
	SEPT8	73300	AT	−1.932573674	0.144775115	0.026213655	0.799576921	0.026657461
	CHCHD3	81833	AT	2.770001828	15.95866318	1.355092185	187.942144	0.027703128
	NRG1	83312	AT	−2.437481846	0.087380613	0.027533352	0.277313543	3.52E-05
	LGALS3	27617	AT	3.525390265	33.96702718	2.214634727	520.970308	0.011382795
	FANCD2	63307	AT	4.12329134	61.76218845	3.510041307	1086.758698	0.004830142
	PSMB7	87532	AT	−2.579926544	0.07577957	0.006666101	0.861454613	0.037506205
ES	NDRG2	26517	ES	1.855248404	6.39328617	1.530859794	26.70009901	0.010963972
	IP6K2	64760	ES	2.795037686	16.3632454	5.32543777	50.27864596	1.06E-06
	MYO19	40481	ES	−1.29099287	0.274997611	0.089421889	0.845695463	0.024299236
	TNFRSF11B	84998	ES	−0.944608524	0.38883176	0.168134419	0.899221814	0.027223571
	CARD8	50713	ES	1.393842997	4.030308793	1.650740804	9.840060248	0.002209495
	CCDC136	81717	ES	1.463262352	4.320030022	1.635017641	11.41434742	0.003159835
ME	DLG3	89383	ME	1.675000904	5.338799977	2.052995404	13.88351145	0.000592306
	SRGAP1	93242	ME	1.803645895	6.0717441	1.260351051	29.25064124	0.024549824
	MAPK10	69825	ME	−3.167912676	0.042091365	0.002548083	0.695300238	0.026832968
	PCBP4	65134	ME	−2.387692442	0.091841369	0.010513434	0.802291368	0.030837457
	RAD51	30020	ME	−3.274891158	0.037820986	0.004606013	0.310556457	0.002299698
	SDR39U1	27012	ME	−1.045775084	0.351419334	0.134765333	0.916374752	0.032472257
RI	NPIPA5	34148	RI	−2.202669028	0.110507816	0.030044922	0.406457286	0.000917135
	MED22	88029	RI	1.960669691	7.104083012	0.867257724	58.19261571	0.067664118
	ABCC5	67820	RI	6.542764727	694.2032097	31.99629894	15061.68251	3.08E-05
	TMEM107	39107	RI	2.317084378	10.1460491	1.982930083	51.91424202	0.005404926
	WDR62	49339	RI	−1.34359868	0.260905063	0.069045763	0.985888914	0.047600621
	GGA3	43399	RI	4.3686116	78.93396369	8.3100749	749.761067	0.000142641
	PPP1CC	24503	RI	−10.12349163	4.01E-05	4.42E-07	0.003642107	1.08E-05
	TEX9	30763	RI	−1.760061746	0.172034241	0.030293932	0.976954054	0.047002651

### Identification of m6A-Related AS events signature independence for prognostic prediction and development of AS-clinicopathological nomogram

**FIGURE 8:**
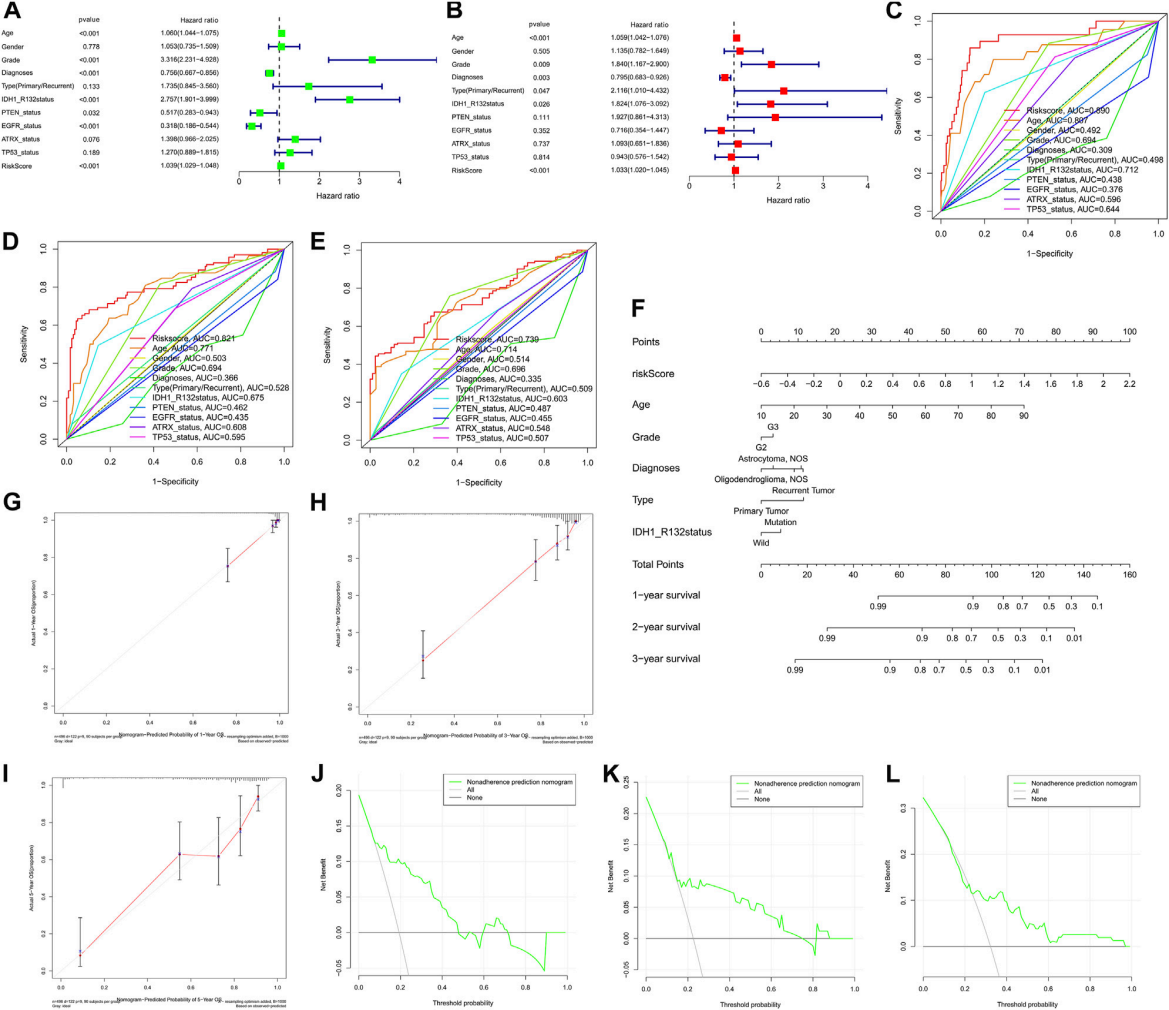
**(A)** nomogram based on the AS signature and clinical characteristics associated with the m6A methylation regulator. Cox regression evaluations on univariate **(A)** and multivariate **(B)** data, as well as the model, indicating excellent prognostic performance independent of clinical variables. **(C–E)**. ROC curve for anticipating overall survival (OS) at 1, 3, and 5 years used to validate the model’s prognostic accuracy in contrast with other individual components. **(F)** A nomogram created using the risk score, grade, diagnoses, age, and type data, as well as the *IDH1* (*R132*) status. **(G–I)**. Calibration plots showing agreement in anticipating the OS at 1, 2, and 3 years. **(J–L)**. The DCA curve demonstrating agreement in anticipating OS at 1, 2, and 3 years.

To explore whether the prognostic significance of *m6A* regulator-associated AS events is independent of clinicopathological variables, we assessed the tumor grade, gender, LGG diagnosis type, age, tumor type (recurrent and primary), as well as the mutation statuses of *IDH1* (*R132*), *ATRX*, *EGFR*, *TP53*, and *PTEN*. and *m6A* regulator-associated AS events based on the univariate and multivariate Cox regression analyses that were conducted for the prognostic risk score model. The findings of the multivariate analysis demonstrated that the risk score was remarkably correlated with OS of patients belonging to the two groups in the TCGA set (*p* < 0.001). In addition, five other clinical factors, namely age, grade, LGG diagnosis type, type (primary and recurrent), and *IDH1* (*R132*) mutation status were also significant (*p* < 0.05) (Table 3 and Figures 8A,B). We also drew ROC curves comparing risk scores with other clinicopathological factors. The findings illustrated that risk score performed better in contrast with tumor grade, gender, age, LGG diagnosis type, tumor type (recurrent and primary), as well as mutation status of *IDH1* (*R132*), *ATRX*, *EGFR*, *TP53*, and *PTEN*, along with some other factors (Figures 8C–E). These findings suggest that the prognostic performance of risk score for survival prediction in LGGs is significantly higher.

**TABLE 3:** Univariate and Multivariate Cox Proportional-Hazards analysis for the N6-Methyladenosine-Related AS Events Riskscore and overall survival in LGG patient cohorts.

Variables		Univariable analysis			Multivariable analysis		
		HR	95%CI	P-value	HR	95%CI	P-value
N6-Methyladenosine-Related AS Events Riskscore	High/Low	1.039	1.029–1.048	<0.001	1.033	1.020–1.045	<0.001
Age		1.060	1.044–1.075	<0.001	1.059	1.042–1.076	<0.001
Gender	Female/Male	1.053	0.735–1.509	0.778	1.135	0.782–1.649	0.505
Grade	G2/G3	3.316	2.231–4.928	<0.001	1.840	1.167–2.900	0.009
Diagnoses	Astrocytoma, anaplastic/Astrocytoma, NOS/Mixed glioma/Oligodendroglioma, anaplastic/Oligodendroglioma, NOS	0.756	0.667–0.856	<0.001	0.795	0.683–0.926	0.003
Type	Primary/Recurrent	1.735	0.845–3.560	0.133	2.116	1.010–4.432	0.047
IDH1 R132status	Mutation/Wild	2.757	1.901–3.999	<0.001	1.824	1.076–3.092	0.026
EGFR status	Mutation/Wild	0.318	0.186–0.544	<0.001	0.716	0.354–1.447	0.352
ATRX status	Mutation/Wild	1.398	0.966–2.025	0.076	1.093	0.651–1.836	0.737
TP53 status	Mutation/Wild	1.270	0.889–1.815	0.189	0.943	0.576–1.542	0.814
PTEN status	Mutation/Wild	0.517	0.283–0.943	0.032	1.927	0.861–4.313	0.111

The nomogram prognostic score system was created with the aim of effectively applying the findings of the present research to clinical practice. It predicts the 1-, 2-, and 3-years OS of patients according to the above-mentioned clinical parameters. This scoring system includes age, tumor grade, LGG diagnosis type, tumor type (primary and recurrence), *IDH1* (*R132*) mutation status, and the risk score of *m6A* regulator-related AS events (Figure 8F). Subsequently, to validate the reliability of this model, we performed calibration plots analysis (Figures 8G–I), and the findings of the present research confirmed the applicability of the model in real-world situations. Hence we validated the data presented above by means of clinical DCA to investigate if nomogram plots could accurately anticipate 1-, 2-, and 3-years OS of patients (Figure 8J–L). The findings revealed that nomogram plots exerted remarkably improved performance in anticipating patient prognosis as opposed to any of the independent variables. To highlight the robust prognostic value of nomogram, we compared the efficacy of other signatures from references. Currently there are only three publications on survival prediction alternative splicing in LGG (Liu et al., 2020; Wang et al., 2020; Zeng et al., 2020). Hence, we used C-index in entire TCGA-LGG cohort to compare the prognostic ability of the different models. C-index value showed that our nomogram had the strongest predictive performance (0.861 > 0.819 > 0.797 > 0.762). In view of the fact that no accurate survival prediction model is available for patients with LGG at present, the development of such a model is of great necessity for both clinical practitioners and patients.

### Relationship between risk score and characterization of the tumor immune environment

**FIGURE 9:**
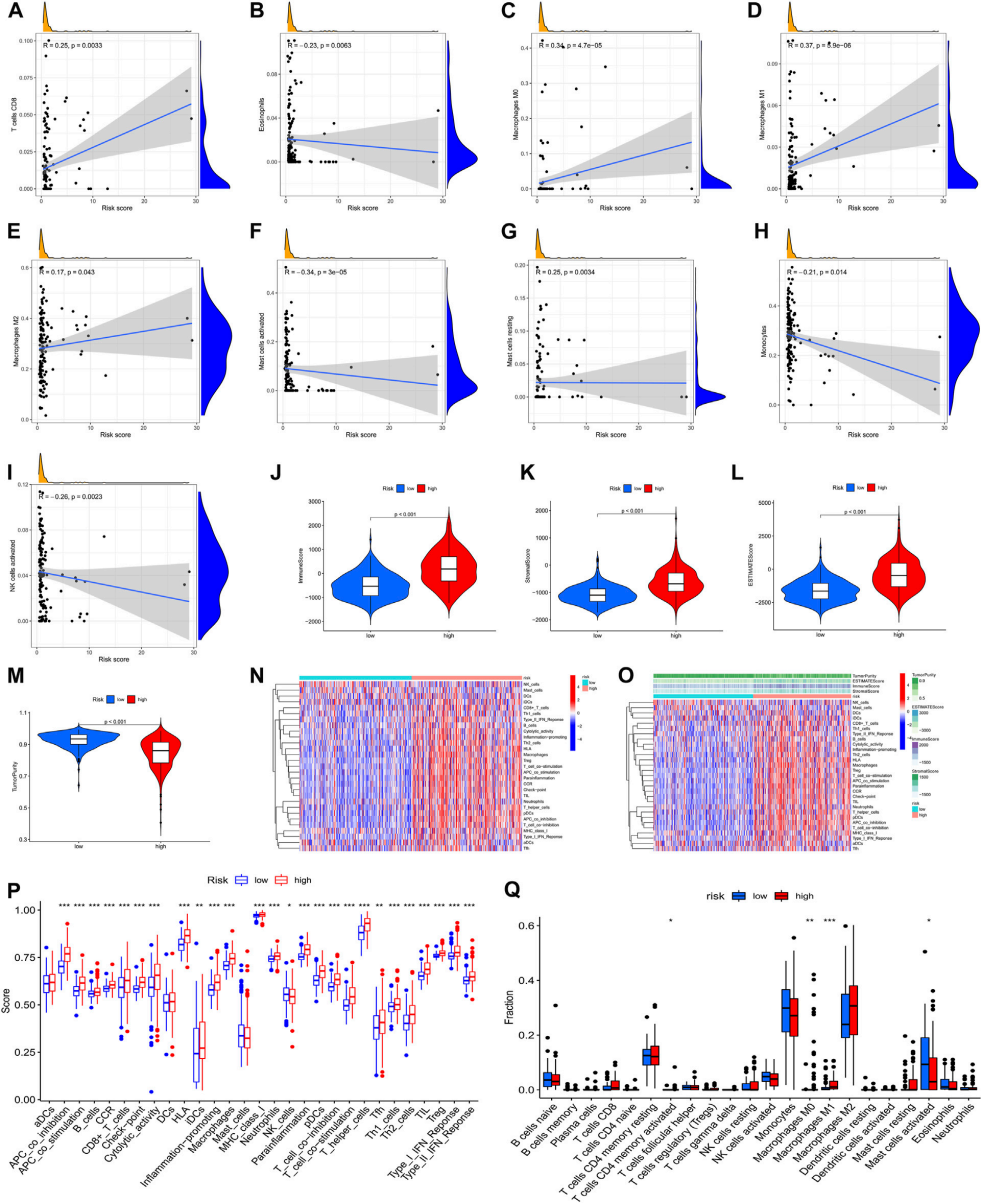
A correlation was observed between the infiltration status of immune cells and an AS-based predictive signature based on the m6A methylation modulator. **(A)**. The correlation between the signature and CD8^+^ T cells. **(B)**. The signature is correlated with eosinophils. **(C)**. The correlation between the signature and M0 macrophages. **(D)**. The correlation between the signature and M1 macrophages. **(E)**. The correlation between the signature with M2 macrophages. **(F)**. The correlation between the signature and activated mast cells. **(G)**. Relationship between the signature and resting mast cells. **(H)**. Relationship between the signature and monocytes. **(I)**. Relationship between the signature and activated NK cells. **(J–M)**. Comparison of ImmuneScore, StromalScore, ESTIMATEScore, and TumorPurity among low- and high-risk groups. **(N)**. Enrichment of 29 immunological markers in low-/high-risk subgroups shown with a heatmap. **(O)**. A heatmap of 29 immunological markers and their associated immune scores for two distinct groups. **(P)**. Differentiation of immunological signature enrichment between the two groups. **(Q)**. Difference of infiltrating immune cell subpopulations and levels between low-/high-risk groups.

To furthermore investigate the potential of the risk score serving as an immune marker, we conducted a correlation analysis between the prognostic risk score and TIMER for TICs, immune score (obtained by the ESTIMATE algorithm), ssGSEA signature, and TIC subtypes and levels (obtained by the CIBERSORT method. Specifically, the findings of the TIMER experiment revealed that the created signatures were correlated with CD8^+^ T cells (*r* = 0.25, *p* = 0.0033), eosinophils (*r =* –0.23, *p* = 0.0063), M0 macrophages (*r =* 0.34, *p* = 4.7e-5), M1 macrophages (*r =* 0.37, *p* = 5.9e-6), M2 macrophages (*r =* 0.17, *p* = 0.043), activated mast cells (*r =* –0.34, *P* = 3e-5), resting mast cells (*r =* 0.25, *p* = 0.0034), monocytes (*r =* –0.21, *p* = 0.014), and activated NK cells (*r =* –0.26, *p* = 0.0023) (Figures 9A–I), indicating that more immune cells were infiltrated in the high-risk samples. Then, we examined the differences in immune scores and infiltration levels of immune cells between the two groups. The ImmuneScore (Figure 9J, *p* < 0.001), StromalScore (Figure 9K, *p* < 0.001), and ESTIMATEScore (Figure 9L, *p* < 0.001) were remarkably elevated in the group at high-risk as opposed to that of the low-risk group. Interestingly, TumorPurity was shown to be elevated in patients belonging to the low-risk group in contrast with those at high risk (Figure 9L, *p* < 0.001). Subsequently, the immune-related characteristics of these two subgroups were distinguished. Figures 9N,O show the immune-associated characteristics of each patient and the matching immune scores for the two subgroups. According to the findings, antigen-presenting cell co-stimulation, antigen-presenting cell co-inhibition, type Ⅱ interferon response, check-point, type I interferon response, inflammation-promoting macrophage, T follicular helper cells, T helper 1/2 cells, para-inflammation, neutrophils, plasmacytoid dendritic cells, T cell co-stimulation, T cell co-inhibition, MHC class I, T helper cells, cytolytic activity, human leukocyte antigen, immature dendritic cells, regulatory T cells, CC chemokine receptor, CD8^+^ T cells, and B cells were all significantly increased with increasing risk scores for immune characteristics (Figure 9P). Further investigation of the changes in immune checkpoint expressions between these two groups was also performed. We observed remarkable differences that were of statistical significance in the expression of checkpoint genes such as activated mast cells, M1 macrophages, M0 macrophages, and CD4 memory activated T cells between the two subgroups (Figure 9Q, *p* < 0.05). In view of the predictive aspects of *m6A* regulator-related AS events, the findings indicate that a novel approach to elucidate the features of immune regulatory networks in LGG could be developed.

### Association between the AS signature and the main molecules in the ICB

**FIGURE 10:**
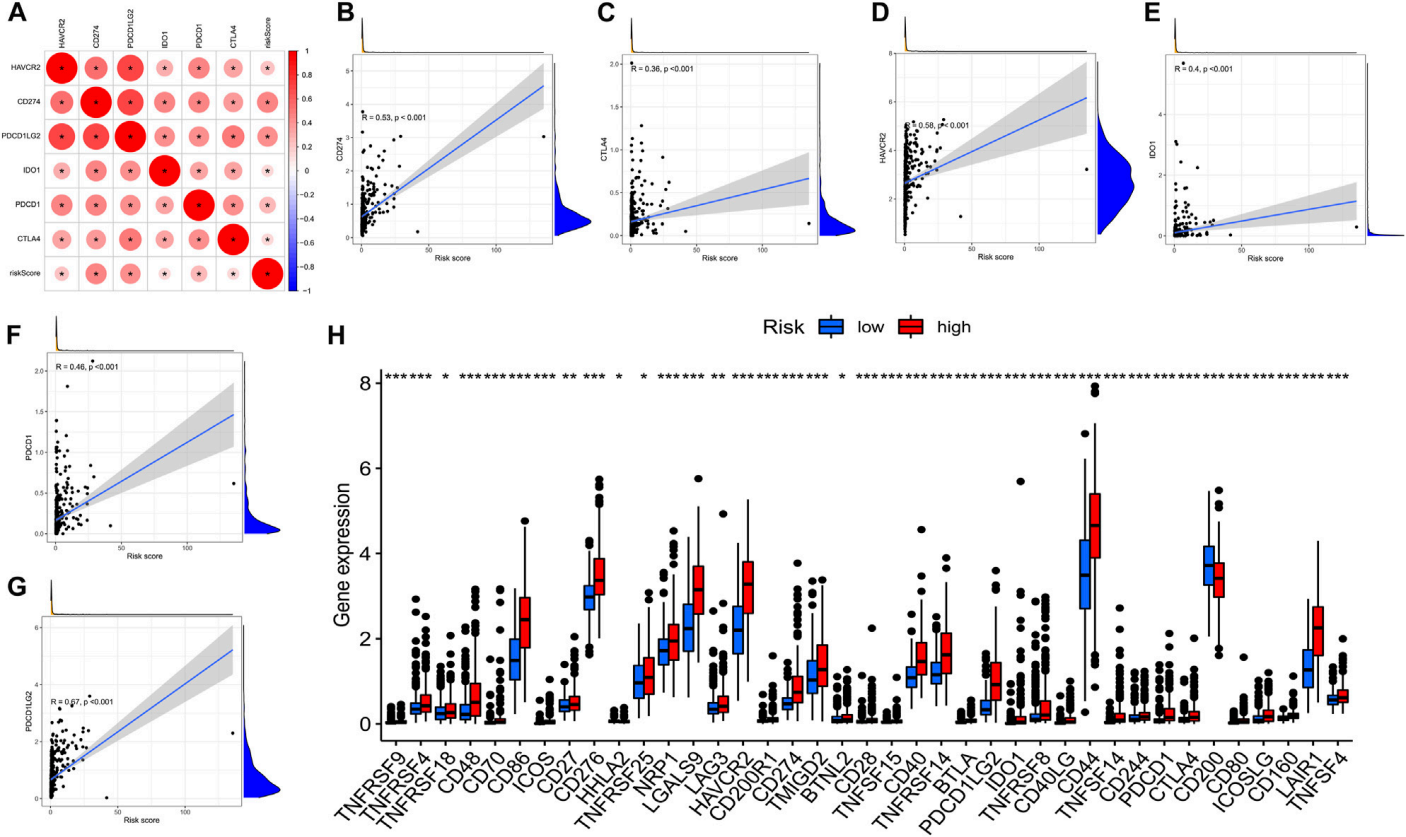
**(A)**. Association between m6A methylation regulator-related AS-based prognostic profile and critical immune checkpoint genes. **(A)**. Analysis of the relationship between the immune checkpoint inhibitors CTLA4, HAVCR2, PDCD1LG2, IDO1, CD274, PDCD1, and the risk score. **(B–F)**. Analysis of the correlation between risk score and PDCD1LG2, CTLA4, HAVCR2, PDCD1, CD274, and IDO1. **(H)**. The gene expression associated with immune checkpoint inhibition between low- and high-risk subgroups.

Immune checkpoint inhibitors, which were introduced with the advent of ICB treatments, have had a significant impact on clinical practice in oncotherapy. Six major immune checkpoint inhibitor genes, including *CTLA*-4, *HAVCR2*, *PDCD1LG2*, *CD274*, *ID O 1*, and *PDCD1*, were shown to be associated with one another. The relationship between crucial ICB targets and prognostic factors of LGG was also investigated for the purpose of determining if risk variables perform a role in ICB for LGG treatment (Figure 10A). The results showed that prognostic features were associated with *CD274* (*r* = 0.53; *p* < 0.001), *CTLA4* (*r* = 0.36; *p* < 0.001), *HAVCR2* (*r* = 0.58; *p* < 0.001), *ID O 1* (*r* = 0.4; *p* < 0.001)), *PDCD1* (*r* = 0.46; *p* < 0.001), and *PDCD1LG2* (*r* = 0.67; *p* < 0.001) (Figures 10B–G). Further correlation analysis demonstrated a remarkable upmodulation in the expression of 39 ICB-related genes in most high-risk patients (Figure 10H), demonstrating that the prognostic features of AS could perform an indispensable function in immunotherapy.

### UGP2 independently affects prognosis and is associated with ICB-related genes and TME

**FIGURE 11:**
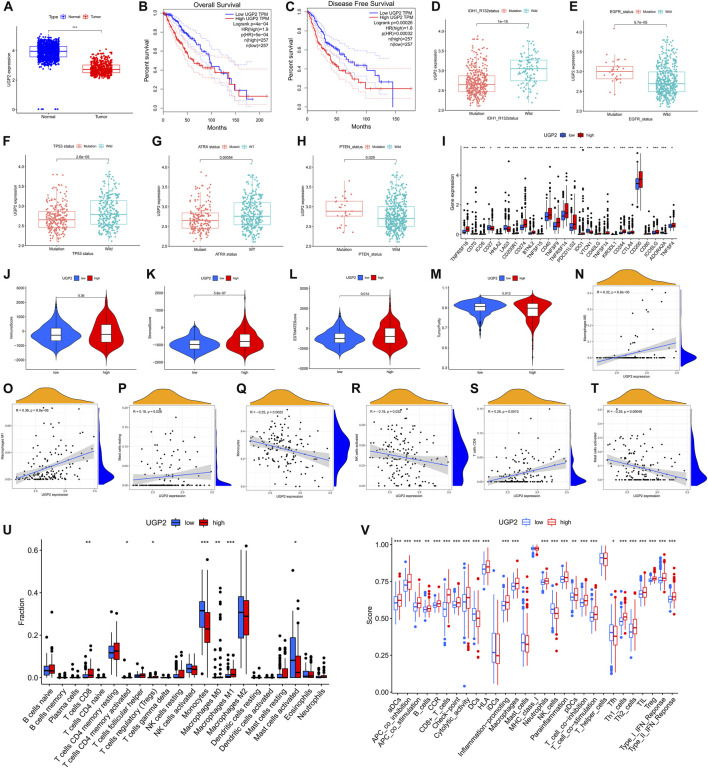
The clinicopathological value of UGP2 in LGG and TME features. **(A)**. *UGP2* expressed in LGG tumor tissue and normal tissue. Lower *UGP2* expression led to prolonged overall survival **(B)** and disease-free survival **(C)**. **(D–H)**. The *UGP2* expression exhibited substantial differences among the mutation status of *IDH1* (*R132*), *EGFR*, *TP53*, *ATRX*, and *PTEN*. **(I)**. The expressions of immune checkpoint blockade-related genes between the low- and high-*UGP2* subgroups. **(J–M)**. Comparison of ImmuneScore, StromalScore, ESTIMATEScore, and TumorPurity among low-/high- *UGP2* groups. **(N–T)**. Relationship between low-/high-*UGP2* groups with activated mast cells, activated NK cells, monocytes, CD8^+^ T cells, resting mast cells, as well as M0 and M1 macrophages. **(U)**. Comparison of CIBERSORT results between low-/high- *UGP2* groups. **(V)**. Comparison of ssGSEA enrichment between low-/high-*UGP2* subgroups.

We performed differential analysis from the AS event genes involved in the model construction, setting a threshold of |logFC|>1 and *p* < 0.05. Ultimately, only two genes (UDP-glucose pyrophosphorylase 2 [*UGP2*], *SDR39U1*) were found to be significantly different from those in tumor and normal tissues. *UGP2* was the primary protein-coding gene as the modification of *UGP2* was the most obvious. Moreover, the expression of *UGP2* is significantly associated with the prognosis of patients with tumors. It has been shown that UGP2 is associated with pancreatic cancer (Wolfe et al., 2021), glioma (Zeng et al., 2019), and other malignancies (Wang et al., 2018). According to the data acquired from the GTEx and TCGA, *UGP2* expression was compared between normal and tumor samples. Relative to tumor tissues, *UGP2* expression was higher in normal samples (Figure 11A). With the aim of fully assessing the predictive utility of *UGP2* in LGG, a Kaplan-Meier analysis was performed on patients having *UGP2*-high- and -low expression through the GEPIA database (http://gepia.cancer-pku.cn/). As shown in Figures 11B,C, lower *UGP2* expression significantly indicated longer OS (*P* = 5e-4) and longer disease-free survival (*p* = 0.00032). Next, we analyzed the *m6A* regulator-associated AS model in different gene mutation and wild-type states and showed that *UGP2* exhibited a remarkable association with the mutation status of *ATRX*, *IDH1R132*, *PTEN*, *EGFR*, and *TP53*(*p* < 0.05) (Figures 11D–H). Moreover, in contrast with the *UGP2*-high and -low groups, the gene expressions in 26 immune check blockage-related genes were significantly dysregulated between the different subgroups (*p* < 0.05) (Figure 11I). We then compared the immune scores between both the *UGP2*-high and -low groups, as well as the infiltration levels of immune cells between the two groups, thus revealing the functions of *m6A* regulator-associated AS events in the immunological milieu of LGGs. StromalScore (Figure 11K, *p* = 5.8e-7) and ESTIMATEScore (Figure 11L, *p* = 0.014) were discovered as being elevated in the *UGP2*-high group in contrast with the -low group, but ImmuneScore was not statistically significant between the two groups (Figure 11J, *p* = 0.36). Interestingly, TumorPurity was remarkably elevated in the *UGP2*-low group as opposed to the -high subgroup (Figure 11M, *p* = 0.012). A correlation analysis was also conducted to examine the relationship between our *m6A* regulator-associated AS prognostic characteristics and the subcategories of immune cell infiltration in LGG. The expression of M0 macrophages, M1 macrophages, resting mast cells, monocytes, activated NK cells, CD8^+^ T cells, and activated mast cells were correlated with risk scores of 0.32, 0.36, 0.18, –0.25, –0.18, 0.26, and –0.29, respectively (Figures 11N–T). The results of CIBERSORT analysis demonstrated that the patients at low risk exhibited a remarkably higher proportion of monocytes, whereas activated mast cells were considerably elevated in patients at low risk. Furthermore, M1 and M0 macrophages, regulatory T cells, memory-activated CD4^+^ T cells, and CD8^+^ T cells were elevated in high-risk patients as opposed to those at low-risk (Figure 11U). The findings recorded from the ssGSEA highlighted that the expression of all immune-associated features was statistically different (*p* < 0.05) between patients with either low or -high *UGP2* expression, except for inhibitory dendritic cells, mast cells, MHC class I, and T helper cells (Figure 11V).

### Analyses of the regulatory network between SFs and AS events utilizing m6A

**FIGURE 12:**
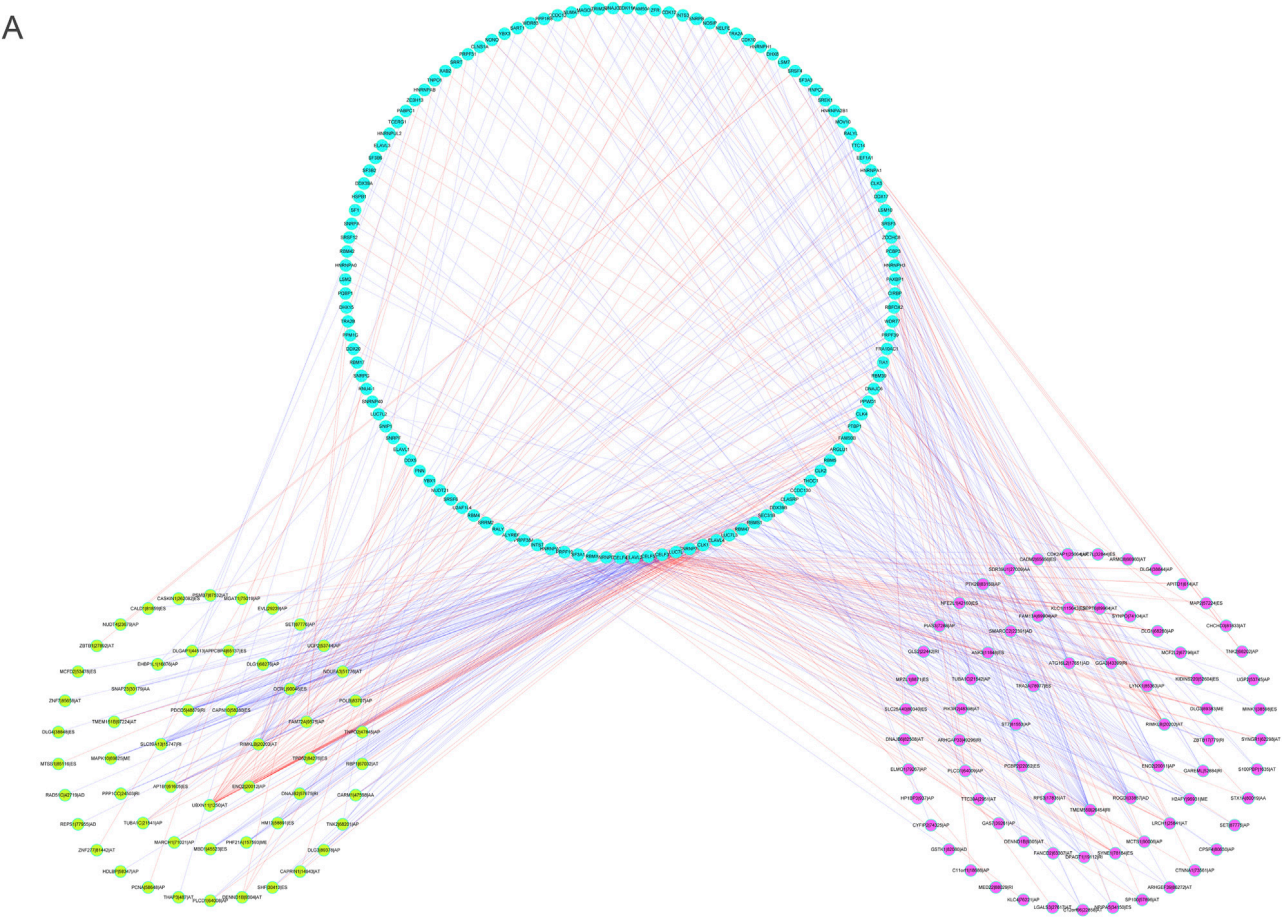
**(A)**. The regulatory network between splicing factors and m6A methylation regulator-associated AS events.

To explore the fundamental processes of AS modulation, we developed a correlation network between the expression patterns of SFs and the PSI values of *m6A* regulator-associated AS events by performing correlation analyses on AS and RNA sequence expression data. In total, 73 upmodulated (purple ovals) AS events, 54 downmodulated AS events (yellow ovals), and 123 SFs (Pearson *r* > 0.6 or < –0.6, *p* < 0.001) were identified (Figure 12A). The aforementioned SFs and AS events yielded 553 SF-AS, 346 of which were positively related and 207 of which were negatively associated. We created a regulatory network using these paired SFs and AS events. Thus, these SFs may be crucial modulators of AS dysregulation in LGG, thus regulating the occurrence and progression of LGG.

## 4 Discussion

An increasing number of researchers are focused on developing AS profiles to assess the prognosis of patients with cancer. However, limited research has been done on the function of *m6A* regulator-associated AS in patient prognosis and the immune microenvironment of malignant tumors including LGGs. Considering the heterogeneity of m6A methylation modifications, it is necessary to quantify the m6A methylation modification profile of individual tumors, such as LGGs. In the present research, we conducted a thorough analysis of the expression, prognostic significance, and impact on the immune microenvironment of m6A methylation-associated AS in LGGs.

We explored the aberrant expression of 12 m6A-regulated genes as well as the AS-related events for the purpose of creating a risk profile that might be employed to anticipate OS in patients with LGG. We found several genes substantially changed in LGG tissues in contrast with their normal counterparts, which may be utilized to anticipate patient survival. First, we discovered that LGG has 48,050 mRNA splicing events and that mRNA splicing can be regulated using the *m6A* regulator. The five AS genes in LGG were employed to create a prognosis-associated AS event signature for LGG, and patients were classified into low- and high-risk groups according to their AS event signature. The data showed that UGP2|53745|AP, SET|87776|AP, SDR39U1|27009|AA, RPAIN|38691|ES, and RAD52|19633|AP were the AS events associated with *m6A* regulators in LGG. Further analysis revealed that risk characteristics, age, tumor grade, LGG diagnosis type, tumor type (primary and recurrent), and *IDH1* (*R132*) mutation status independently served as prognostic indicators of LGG. In addition, the AUC of the ROC curves demonstrated better specificity and sensitivity of LGG than other recent studies, respectively (Wang et al., 2020). In conclusion, the present research demonstrates the role of this AS prognostic feature in predicting LGG prognosis. The validity of this AS feature will be confirmed in future research with the aid of a prospective dataset of patients with LGG.

Various treatment methods, such as histone deacetylase inhibitors and drugs targeting hypoxia-related pathways, have been developed as a result of substantial research into the role of DNA and epigenetic histone modifications in cancer progression (Miller et al., 2009). However, the specific role of mRNAs in different cellular processes has become a rapidly developing field in the last decade (Ge et al., 2020). At present, M6A is the most prevalent type of mRNA modification in eukaryotes and abundant total adenosine has been found in 0.1–0.4% residues (Rauch et al., 2018). M6A has already been demonstrated to be common across the transcriptome, and it has been found in the mRNAs of over 7,600 genes in addition to more than 300 non-coding RNAs(Lu et al., 2020). It is an evolutionarily conserved gene in both humans and mice located close to the stop codon in the 3′UTR as well as in internal exons of both species (mostly variable exons); moreover, it can alter RNA stability, AS, intracellular distribution, and translation (Niu et al., 2020). AS is an extremely common process where a single pre-mRNA may lead to several mature mRNAs, thus increasing the variety of proteins and enabling the cell to become more complex in terms of both regulatory and functional complexities (Wang et al., 2021). According to genome-wide research, 90–95% of human genes are subjected to some kind of AS at some timepoint, and ∼1/3 of these genes (including m6A-related genes) have been shown to produce multiple protein isoforms (Chen X. et al., 2021). Studies have shown that AS has an integral function in the acquisition of tissue properties, organ development and growth, as well as being involved in a wide range of pathological changes, such as cancer (Wang et al., 2021). Human cancers can use paradoxical AS for their occurrence, growth, and development into treatment-resistant cancers. Wu et al. revealed METTL3-D splice variant is a tumor suppressor that could potentially be used as a target for hepatocellular carcinoma therapy (Xu et al., 2022). Wang et al. establishes a link between SRSF3, m6A modification, lncRNA splicing, and DNA HR in pancreatic cancer and demonstrates that abnormal alternative splicing and m6A modification are closely related to chemotherapy resistance in pancreatic cancer (Wang et al., 2022).

M6A modifications and AS are the most common types of alterations found in mRNA transcripts, and these alterations are thought to have a significant impact on the occurrence and progression of human malignancies (Makhafola et al., 2020). It has demonstrated that gene methylation facilitated by the *m6A* regulator is essential for the occurrence and progression of LGG. Nevertheless, there has been slow progress to date in comprehending the possible molecular pathways underlying the role of *m6A* regulation in cancer progression. During AS events, we discovered that *m6A* regulators perform fundamental functions as SFs. To further investigate *m6A* regulators and associated AS events in LGGs, we performed GO term and KEGG pathway analysis of the remarkable enrichment of *m6A* regulators in biological processes such as mRNA spliceosome biological processes, RNA modification (RNA methylation biological processes), and RNA instability. It was found that *m6A* regulators influence the course of AS events. For example, AS of VEGFA, as well as osteogenic differentiation of bone marrow mesenchymal stem cells, are regulated by the transcription factor *METTL3* (Tian et al., 2019). In human pulp cells, the *m6A* writer *METTL3* modulates the AS of MyD88 in responses to lipopolysaccharide-elicited inflammation (Feng et al., 2018). Furthermore, in the vicinity of the AS exon and poly-A sites, the m6A demethylase FTO has the ability to target pre-mRNAs and regulate AS and 3′ end processing. The AS effect of *FTO* knockdown is negatively associated with *METTL3* knockdown, demonstrating the role of m6A (Bartosovic et al., 2017). Mettl3-mediated m6A modulates the differentiation of spermatogonia, initiation of meiosis, as well as differences in gene expression that participate in spermatogenesis and the spectrum of AS (Xu et al., 2017). METTL3 has also been shown to interact with skipped exons and AS events that substitute for the first exon (Mauer et al., 2019). Moreover, they found that the WTAP complex regulates AS of WTAP pre-mRNA by enhancing the synthesis of shortened isoforms, leading to alterations to the expression of the WTAP protein (Horiuchi et al., 2013). Tang and Klukovich also discovered that m6A demethylation that is dependent on ALKBH5 affects the stability and splicing of long 3′-UTR mRNA in male germ cells (Tang et al., 2018). A new stage in snRNA processing that included reversible methylation was discovered to be regulated by FTO, indicating that the epigenomic information contained in snRNA may have an impact on the AS patterns (Mauer et al., 2019). Luxton et al. discovered that the oncogene *metadherin* interacts with known splicing proteins T-STAR, Sam68, and YTHDC1, while also performing an instrumental function in alternative mRNA splicing (Luxton et al., 2019). During the course of mouse oocyte growth, the nuclear *m6A* reader YTHDC1 has been shown to modulate alternative polyadenylation and shearing. Furthermore, YTHDC1 deficiency results in a greater proportion of AS defects in oocytes (Kasowitz et al., 2018). This suggests that the *m6A* reader YTHDC1 affects AS processes. According to the findings of Fischl, hnRNPC modulates cancer-specific alternative polyadenylation and cleavage (Fischl et al., 2019). Clearly, the *m6A* RNA methylation regulatory genes mentioned above are critical in the regulation of AS events in LGG.

The present research demonstrated that AS events are a crucial mRNA modification process, as they result in the generation of a wide scope of mRNA and protein isoforms with a variety of modulatory roles. AS events in LGG have been shown to have prognostic significance, as demonstrated by Wang et al. who established a prediction model for abnormal AS events and anticipated the prognosis of patients with LGG (Wang et al., 2020). Specifically, the alternation of SF expression can influence numerous AS events in tumors. It was found that isoforms of the metabolism-related gene *UGP2* may perform an instrumental function as an AS factor in HCC(Li S. et al., 2019). It was also found that hTERT+β was shown to correlate with clinical characteristics of glioma and might be used as a prognostic indicator or possible treatment target for glioma. CX-5461 can alter the splicing sequence of hTERT, thus suppressing the action of telomerase, and destroying GBM cells (Li et al., 2018).

In addition, we conducted GO and KEGG pathway enrichment analyses with the aim of discovering genes that were substantially implicated in m6A-related AS events in gene pathways involved in LGG tumorigenesis, progression, and metastatic processes. The findings reported from the KEGG analysis illustrated that genes in m6A-related AS events remarkably participate in MAPK signaling in LGG, which has a three-stage signaling process: MAPK, MAPK kinase (MKK or MEK), and kinase of MAPK kinase (MEKK or MKKKK) (Naik et al., 2017). The 3 kinases are triggered sequentially and jointly modulate a wide range of physiological functions, including inflammation, apoptosis, cancer progression, tumor cell invasion, and metastasis (Haagenson and Wu, 2010). They are activated by a series of extracellular stimulatory signals and mediate signal transduction from the cell membrane to the nucleus. However, further research is warranted to confirm this conjecture.

In order to determine the significance of AS events in LGG, we performed a CIBERSORT analysis and used the ssGSEA approach, the ESTIMATE algorithm, as well as the TIMER database. These findings indicate that the high-risk score group had a greater infiltration score of immune cells as well as a more active immunological profile, which might also enhance immune identification and activate anti-tumor activities in the tumor cells. Such findings indicated that risk scores might participate in the prediction of immunotherapy outcomes. Subsequently, we showed that the risk score was strongly associated with the expression of six ICB targets (i.e., *CTLA4*, *HAVCR2*, *PDCD1G2*, *ID O 1*, *PDCD1*, and *CD274*) and 39 immune check blockage-related genes (e.g., *TNFRSF9, UGP2*) implying that the risk score may help in developing targeted immunotherapy strategies. UGP2, the enzyme encoded by this the bridge of the ICB targets and immune check blockage-related gene is an important mediator of mammalian carbohydrate interconversion. It achieves this by transferring a glucose group from glucose-1-phosphate to MgUTP, contributing to the formation of UDP-glucose and MgPPi(Wolfe et al., 2021). Zeng et al. found that UGP2 was identified as a progression marker that promotes the growth and motility of human glioma cells and performs a crucial role in the proliferation of LGG (Zeng et al., 2019). Hu et al. found that low *UGP2* expression was associated with tumor progression and poor prognosis for HCC(Hu et al., 2020). Nonetheless, studies on the function of UGP2 in tumors are limited, particularly in LGG. The present study showed that low expression of *UGP2* was associated with better OS and DFS compared to high expression. *UGP2* expression correlated significantly with the mutation status of *IDH1* (*R132*), *ATRX*, *EGFR*, *TP53*, *PTEN*, and most immune checkpoint genes. Nonetheless, more detailed research is necessary for the purpose of examining the potential biological function of UGP2. Given that the risk score correlates with the ICB targets expression, it can be hypothesized that the antitumor effects of immune cells could be influenced by the ICB pathway.

In contrast with existing studies exploring new prognostic factors in LGG, our study has several highlights. Firstly, the present research makes a significant contribution to the exploration of the possible significance of m6A regulator-associated AS events in the creation of LGG TME complexity and diversity, as well as in the anticipation of ICB therapeutic efficacy, which had not previously been explored. In addition, extensive analyses were performed, including WGCNA, six machine learning algorithms (boosting, bagging, XGBoost, Adaboost, GBDT, randomforest), TIMER database, CIBERSORT method, ssGSEA algorithm, and ESTIMATE R package to reveal the integrated landscape of LGG. Moreover, as far as we know, the present research is the first to highlight the biological function of UGP2 in LGGs. We do, however, acknowledge that there are several limitations to the present research, such as the relative simplicity of the AS event database and the absence of all other pertinent datasets to corroborate our findings. Furthermore, there has been little investigation into the correlation between m6A regulators and AS events, as well as the processes through which they contribute to the occurrence and progression of LGG. As a result, further research is required so as to reveal the real biological significance of AS events in the occurrence and progression of LGG.

However, there are still some limitations of our study that deserve to be stated. Firstly, alternative splicing is different from qPCR or gene knockdown, which requires ultra-deep WES sequencing of clinical samples, yet we currently do not have sufficient funding for this study. Therefore, it may be difficult to carry out relevant experimental verification. In addition, currently only the TCGA project has funding for ultra-deep whole exon sequencing to identify alternative splicing events. All other databases don’t contain alternative splicing data. Therefore, this study only carried out internal validation. In conclusion, a systematic analysis of the prognostic predictive significance of *m6A* modulator-associated AS shear patterns was performed to enhance the prognostic prediction of LGG. Notably, we established new and robust prognostic nomograms to quantitatively predict outcomes, which showed encouraging potential in clinical applications. In addition, the AS-SF regulatory network provides a good target for the antitumor treatment of LGG. Our study provides novel insight into the function of AS in m6A methylation and reveals potential mechanisms by which *m6A* regulator-associated AS events affects tumor progression in LGG.

## Data Availability

The datasets presented in this study can be found in online repositories. The names of the repository/repositories and accession number(s) can be found in the article/supplementary material.
